# Empowering digital health management with on-device large language models for glucose prediction: a model development and validation study

**DOI:** 10.1016/j.ebiom.2026.106343

**Published:** 2026-06-25

**Authors:** Taiyu Zhu, Joanna Howson, Alejo Nevado-Holgado

**Affiliations:** a Department of Psychiatry, University of Oxford, Warneford Hospital, Oxford, OX3 7JX, UK; b Department of Biostatistics & Health Informatics, Institute of Psychiatry, Psychology & Neuroscience, King's College London, 16 De Crespigny Park, London, SE5 8AB, UK; c Novo Nordisk Research Centre Oxford, Old Road Campus, Roosevelt Drive, Oxford, OX3 7FZ, UK

**Keywords:** Continuous glucose monitoring, Deep learning, Digital health, Glucose prediction, Large language models, On-device inference

## Abstract

**Background:**

Long-term management of chronic diseases such as diabetes is increasingly based on wearable technologies, particularly continuous glucose monitoring (CGM), integrated with smartphone-based digital health systems. When combined with artificial intelligence, especially deep learning, these systems offer highly personalised decision support, including glucose prediction. Although large language models (LLMs) have demonstrated strong performance across various healthcare tasks, their integration into day-to-day digital health remains limited, primarily due to privacy concerns associated with transmitting sensitive data to remote servers. Recent advances in lightweight LLMs create new opportunities for secure and local deployment.

**Methods:**

In this study, we first evaluated the zero-shot glucose prediction performance of eight pretrained lightweight LLMs across multiple model families. None achieved clinically viable outputs, highlighting the need for domain-specific adaptation. To address this, we propose GluLLM, a multimodal adaptor-based framework that enhances pretrained LLMs for on-device glucose forecasting. GluLLM integrates CGM data, daily activity logs, and electronic health records using customised encoder and decoder modules while preserving the foundational capabilities of pretrained LLMs. We trained and evaluated GluLLM on the REPLACE-BG dataset, which includes 226 individuals with type 1 diabetes, and validated it on an external cohort comprising 207 individuals with type 2 diabetes or without diabetes.

**Findings:**

Compared with 15 state-of-the-art deep learning baselines for time-series prediction, GluLLM (LLaMA 3.2 1B backbone) demonstrated superior performance, with significantly lower 30-min root mean square error than the strongest baseline (Crossformer) on REPLACE-BG and Móstoles (20.6 ± 3.5 and 9.6 ± 2.9 mg/dL; *p* < 0.001), and improved hypoglycaemia prediction (glucose <70 mg/dL; AUROC: 0.79 and 0.84; AUPRC: 0.55 and 0.60), respectively. Furthermore, deployment of GluLLM on two smartphone platforms demonstrated feasible computational requirements, with acceptable CPU and memory usage and low inference latency.

**Interpretation:**

GluLLM demonstrates that LLMs can support the next generation of smartphone-based digital health systems, delivering real-time, privacy-preserving clinical decision support.

**Funding:**

Novo Nordisk Postdoctoral Fellowship run in partnership with the University of Oxford.

**Research in context**

**Evidence before this study**

We searched PubMed for studies published between 1 January 2015 and 31 May 2026 without language restrictions. The query (“continuous glucose monitoring” OR CGM) AND (prediction OR forecasting) AND (“deep learning” OR “machine learning” OR “artificial intelligence”) yielded 263 articles. When the model's technical terms were replaced with (“large language model” OR LLM OR GPT), the search yielded five articles. These included studies applying large language models (LLM)-based approaches to continuous glucose monitoring (CGM) querying, CGM data summarisation, conversational diabetes management, or glucose forecasting in type 1 diabetes. However, we did not identify a study evaluating a fully smartphone-resident LLM framework, or one that integrates CGM, insulin events, and demographic prompts for glucose prediction. A separate search combining (“digital health”) AND (“on-device” OR edge OR smartphone) AND (“large language model” OR LLM OR GPT) yielded seven articles. These studies were not centred on glucose prediction; where mobile or edge deployment was considered, LLMs were generally accessed through external services rather than local deployment, proposed as future work, or evaluated for non-wearable clinical tasks.

**Added value of this study**

This study demonstrated that compact, multimodal adaptors can repurpose pretrained LLMs for accurate glucose prediction while running entirely on a smartphone. GluLLM combines CGM data, insulin events, and demographic prompts within a frozen LLM backbone and outperforms various deep learning baselines across two independent cohorts. It also operates within practical CPU and memory limits on both mainstream and resource-constrained smartphones, demonstrating that real-time, privacy-preserving decision support is possible without relying on cloud resources.

**Implications of all the available evidence**

Edge-deployable LLMs such as GluLLM could enable secure, personalised clinical decision support and proactive alerts directly on users’ smartphones, reducing reliance on remote servers and mitigating privacy concerns. The same adaptor strategy may generalise to other chronic disease sensors and multimodal data streams. Prospective trials are needed to evaluate clinical impact, user adherence, and regulatory considerations for widespread on-device LLM deployment in digital health.

## Introduction

Diabetes, resulting from inadequate insulin secretion or beta cell dysfunction, affects more than 500 million people worldwide, with approximately 90% classified as type 2 diabetes (T2D) and 10% as type 1 diabetes (T1D).^1^^,^^2^ Suboptimal glycaemic control is associated with cardiovascular, neurological, and retinal complications,^3^ which require frequent glucose monitoring and repeated insulin administration. Continuous glucose monitoring (CGM) has transformed care with minimally invasive sensors measuring interstitial glucose every 5 min, providing real-time blood glucose estimates and improving outcomes in T1D and T2D.^4^^,^^5^ Modern CGM systems provide trend arrows and alerts intended to support glucose management. Trend arrows primarily summarise the near-term direction, typically estimated from recent filtered CGM readings and mapped to discrete categories. Alerts are commonly implemented using high and low glucose thresholds and rapid rate-of-change rules (rise or fall), and some platforms additionally provide predictive low-glucose warnings based on short-horizon prediction. For example, Dexcom G6's alert “Urgent Low Soon” can be triggered when glucose is predicted to reach ≤ 55 mg/dL within 20 min.^6^ However, physiological delays constrain the intervention. There is a notable time delay of five to 10 min between changes in blood glucose levels and their detection by interstitial CGM sensors.^7^ Consequently, timely detection of hypoglycaemia is a major challenge for CGM-based care because episodes are often asymptomatic and can progress to life-threatening events such as seizures, loss of consciousness, and stroke.^8^ These temporal dynamics underscore the importance of proactive diabetes management, for which accurate longer-horizon glucose prediction constitutes a key foundation for timely and effective intervention.

Reliable glucose prediction is hindered by large inter- and intra-individual variability.^9^ Daily factors, such as food, insulin, activity, and individual characteristics, such as HbA1c and body mass index (BMI), strongly influence glucose dynamics.^10^ Recent advances in artificial intelligence (AI), particularly deep learning,^11^ have enabled the effective use of CGM time series. In industry, AI-enabled predictive features are also emerging. For example, Roche's Accu-Chek SmartGuide Predict app received CE marking in 2024 and is described as providing hypoglycaemia risk prediction within the next 30 min in people with T1D and T2D.^12^ In the literature, a variety of deep neural network architectures designed for sequence modelling have been proposed to address this task, including convolutional neural networks (CNNs),^13^ recurrent neural networks (RNNs) such as long short-term memory (LSTM)^14^ and gated recurrent unit (GRU)^15^ models, and more recently Transformer-based models.^16^ These deep learning algorithms predict glucose levels 30–60 min in advance, allowing sufficient time for proactive interventions and achieving strong predictive accuracy, as quantified by metrics such as the root mean square error (RMSE).^11^ Among current approaches, there is a critical trade-off between personalised and population models. Here, we use personalised to describe approaches that adapt a model to an individual using subject-specific data. Many CGM prediction studies use deep learning architectures with individual-specific parameters learnt on extended personal CGM histories.^14^^,^^15^ While such personalisation can improve accuracy, it typically requires a run-in period of weeks to months to accumulate sufficient individual CGM data to learn subject-specific model parameters. This requirement can delay delivery of clinically useful predictions and limit scalability in real-world deployment, where users and services cannot reasonably wait for prolonged data collection before benefiting from decision support. In addition, when models are updated for each new individual, governance and regulatory requirements become more complex, including individual-dependent model updates and post-deployment surveillance. Population models offer practical alternatives, but struggle with inter-subject variability. Furthermore, most existing approaches adopt a single prediction horizon (e.g., predicting glucose only 30 min ahead), rather than forecasting a trajectory over multiple future time points.^11^^,^^14^^,^^15^ This single-horizon formulation can limit clinical decision support because it provides only one point estimate and does not characterise how glucose is expected to evolve across the near-term window relevant to treatment decisions.^17^

Recently proposed large language models (LLMs) offer promising directions for integrating complex clinical knowledge.^18^ LLMs have demonstrated strong zero-shot and few-shot performance on a range of clinical tasks without task-specific fine-tuning, as shown on medical question-answering and clinical reasoning benchmarks.^19^^,^^20^ LLMs also demonstrate state-of-the-art performance in time series applications^21^^,^^22^ through learnt token transitions and language-based embeddings. Despite this success, the applications that use CGM data for glucose prediction or analysis remain limited. Relevant studies remain sparse, including one evaluating TimeLLM for personalised glucose prediction,^23^ one using GPT-4 for CGM data analysis,^24^ and a recent study evaluating Chronos and TimeLLM forecasting for glucose prediction.^25^ A key challenge in LLM-driven healthcare is addressing security and privacy concerns with remote processing.^18^ This sparked the development of smaller and more efficient LLMs for local deployment. Apple launched on-device LLMs (July 2024) with API access (June 2025), followed by Meta's LLaMA 3.2 edge models (September 2024). This aligns with current CGM practice, in which smartphone apps support data visualisation and clinical decision support,^26^ including the deployment of predictive algorithms such as deep learning models for glucose prediction.^15^ Recent progress in LLMs for healthcare has also increasingly emphasised small LLMs and parameter-efficient adaptation to enable deployment in resource-constrained and privacy-sensitive settings. Domain-specialised biomedical models such as BioGPT^27^ demonstrate that competitive biomedical language understanding can be achieved with substantially fewer parameters than general-purpose LLMs. Efficient training and adaptation approaches, including improved in-context learning strategies (e.g., SuperICL^28^) and parameter-efficient methods (e.g., MedAdapter^29^), further reduce the compute and memory required to tailor models to clinical tasks. Leveraging this momentum, we introduce GluLLM, a smartphone-based, LLM-driven framework designed for secure, real-time glucose prediction. GluLLM supports personalisation without accumulating individual-specific CGM data and enables multi-horizon forecasting up to 1 h through efficient training of lightweight adaptor modules. Our primary benchmarking evaluates GluLLM against established machine learning and deep learning baselines for CGM time-series prediction, rather than framing the study as a competition among different foundation model backbones.

## Methods

### Clinical data preprocessing

This study used de-identified data from two previously approved cohorts (Supplementary Table S1). REPLACE-BG^30^ contains data from 226 T1D participants across 14 clinical sites using Dexcom G4 CGM for 26 weeks. The Móstoles dataset^31^ comprises 207 participants with hypertension (no prior diabetes) monitored with Medtronic iPro devices for 24–72 h, with 17 developing T2D during 33-month follow-up. Both datasets provide demographic variables and CGM measurements at 5-min intervals, with a balanced sex distribution (male: 50.4% in REPLACE-BG and 49.8% in Móstoles). Sex was recorded as a baseline demographic characteristic at enrolment or recruitment in the source studies and used here as provided in the original datasets. REPLACE-BG includes insulin bolus data, which is absent from the Móstoles dataset. Supplementary Table S2 shows significant differences in mean glucose and time in target range (TIR), underscoring the importance of model generalisation capabilities for effective cross-dataset performance.

**Fig. 1:**
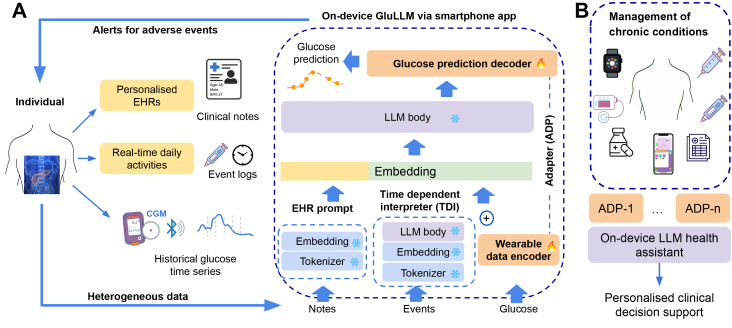
Integration of LLMs in chronic disease management through adaptive frameworks. **A.** Implementation of GluLLM on smartphone platforms that seamlessly integrate with digital health ecosystems for comprehensive glucose management. The system processes CGM data transmitted via Bluetooth, captures daily activities through event logs, and accesses electronic health records (EHRs) stored locally. All data streams are processed through specialised components of the GluLLM architecture, including EHR prompt, time-dependent interpreter (TDI), and wearable data encoder, to deliver personalised insights and predictions. In the schematic, ice icons indicate preserved pre-trained LLM elements that remain frozen, while fire icons denote adaptor components requiring targeted training. **B.** Extensibility of the framework through multiple specialised adaptors (ADPs), each equipped with dedicated encoder and decoder modules for handling diverse data types. This design enables management of multiple chronic conditions through a unified on-device LLM platform.

We utilised all available information in this real-world glucose-management setting (Fig. 1) and distinguished between static or slowly varying clinical context and time-stamped dynamic events. Demographic and clinical variables derived from the EHR (e.g., age, sex, BMI, HbA1c) change infrequently relative to CGM sampling and were therefore encoded as a structured EHR prompt to provide global conditioning for personalised prediction. All participants in REPLACE-BG had complete demographic and clinical variables. In the Móstoles cohort, BMI was missing for one participant and HbA1c for eight participants; these missing values were encoded as “Unknown” in the EHR prompt. In contrast, event logs (e.g., insulin delivery, meals, and activity) are time-dependent exposures whose effects depend on their timing relative to the CGM trajectory. We therefore encoded events using the time-dependent interpreter (TDI) and passed the resulting representations through the LLM backbone to learn temporally aligned features that can interact with the CGM token sequence and capture event–glucose dependencies.

Demographic information was extracted and formatted as an EHR prompt: “Characteristics: Age: {age}; Sex: {sex}; BMI: {bmi}; HbA1c: {hba1c}. Predict the next values based on the historical glucose embedding.” Timestamped CGM data were formatted in date–time form and, when available, combined with insulin bolus information as input to the TDI module, for example: “Historical data from 13:30 to 14:30; 5 units of bolus insulin delivered.” For datasets without bolus insulin records, the TDI input included only the corresponding time-window description (e.g., “Historical data from 13:30 to 14:30.“). CGM values were standardised before modelling. To prevent data leakage during imputation, missing values within a sequence were filled by linear interpolation, whereas missing values at sequence boundaries were handled using extrapolation. The wearable data encoder then ingested an observation window of length L at a timestep t, denoted $G_{t}=\left[g_{t-L+1},g_{t-L+2},\ldots ,g_{t}\right]\in R^{L}$. The model predicts the subsequent glucose trajectory over a horizon τ, ${\hat{G}}_{t}=\left[{\hat{g}}_{t+1},{\hat{g}}_{t+2},\ldots ,{\hat{g}}_{t+\tau }\right]\in R^{\tau }$.

### GluLLM for multimodal data integration

We employed LLM tokenizer and embedding modules to convert EHR prompts into embeddings **EE**, with frozen parameters that require no training. Since demographics remain constant per individual, this operation occurs once. The historical glucose **G**_*t*_ was then converted to language-compatible embeddings. Various methods exist in the literature for this dimension alignment, ranging from simple linear layers to more complex multi-head attention mechanisms.^21^ Inspired by a recent efficient approach proposed in AutoTimes,^22^ which effectively captures rich chronological information, we segmented CGM data into tokens, each converted by GluLLM's wearable data encoder to match language token dimensions using a fully connected network (Supplementary Fig. S1). Although the length of the segments could be arbitrary, we used the prediction length *τ* as the segment length to naturally reformulate the problem into next-token prediction, which is a standard training objective for decoder-only LLMs.^32^ This approach allowed us to leverage the powerful predictive capabilities and token transition inherent in these language models. Additionally, we assumed that the length of historical CGM data L was N multiples of τ to ensure consistent and uniform segmentation throughout the analysis. In this case, the input and output for glucose prediction are $G_{t}=\left[k_{1}^{T},k_{2}^{T},\ldots ,k_{N}^{T}\right]\in R^{\tau \times N}$, ${\hat{G}}_{t}={\hat{k}}_{N+1}^{T}$, where $k_{n}=\left[g_{t+\left(n-N-1\right)\tau +1},g_{t+\left(n-N-1\right)\tau +2},{\ldots ,g}_{t+\left(n-N-1\right)\tau +\tau }\right]$. Here, we set L=72 and τ=12, corresponding to 6 h of historical CGM readings and a prediction horizon of 1 h. The 6-h window captures the typical duration of insulin action, as the effects of a meal insulin bolus generally subside within this period.

After converting each token **k**, we obtain the glucose embedding:

(1) $${\text{GE}}_{t}=\text{GluLLMEncoder}\left(G_{t}\right)=\left[{\text{ge}}_{1}^{T},{\text{ge}}_{2}^{T},\ldots ,{\text{ge}}_{N}^{T}\right]\in R^{d\times N}\text{,}$$

where *d* denotes the dimensionality of the LLM's embedding, which varies across the different models considered in this work. To fully leverage temporal information, we also divided the timestamp and insulin data into the same segments used for the CGM data, and processed them in the TDI. As illustrated in Fig. 1, TDI comprises a tokenizer, an embedding layer, and the LLM body from the original LLM. This design allows the model to process temporal information and associated daily activities, producing a corresponding language embedding. Specifically, we selected the last hidden state vector from the output embedding of each segment, as this position typically aggregates the most relevant contextual information from the input text. By concatenating these vectors, we obtain ${\text{TE}}_{t}=\left[{\text{te}}_{1}^{T},{\text{te}}_{2}^{T},\ldots ,{\text{te}}_{N}^{T}\right]\in R^{d\times N}$. Aligning the temporal embedding vectors with the glucose embedding vectors in a one-to-one fashion, similar to positional encoding, and prepending the EHR prompt embedding at the beginning, we construct the final input embedding for the LLM. The output embedding is then obtained by processing this input through the LLM body:

(2) $${\text{OE}}_{t}=\text{LLMBody}\left(\text{Concat}\left(\text{EE},\left({\text{GE}}_{t}+{\text{TE}}_{t}\right)\right)\right)\text{,}$$

where the addition is performed element-wise. In this regard, the next hour of glucose levels can be obtained by projecting the last hidden state using the GluLLM decoder, which is also implemented as a fully connected neural network (Supplementary Fig. S1), and we have

(3) $${\hat{G}}_{t}=\text{GluLLMDecoder}\left({\text{OE}}_{t}^{-1}\right)\text{.}$$

### Model training and evaluation

To train a population-level glucose prediction model, we used a participant-disjoint split, assigning each individual's complete data record to only one of the training, validation, or test sets so that evaluation reflects performance on unseen individuals. Given the substantial volume of data in the REPLACE-BG dataset (approximately 12 million CGM readings), we used it for model training, validation, and hold-out testing, while the Móstoles dataset served as an external validation cohort. Specifically, the REPLACE-BG dataset was first divided into a development set comprising 180 individuals and a hold-out testing set of 46 individuals. We then performed five-fold cross-validation on the development set to fine-tune the hyperparameters of GluLLM. In each fold, data from 144 individuals were used for training, while the remaining 36 individuals were used for validation. This process was repeated across folds to ensure robust hyperparameter selection (Supplementary Fig. S2); the full list of hyperparameters is provided in Supplementary Table S3. Notably, hyperparameter tuning was performed for all baseline models to ensure fair comparisons.

The performance of the model was evaluated using RMSE, reflecting the overall prediction error, with global RMSE measuring the error throughout the prediction horizon *τ*. Mean absolute error (MAE) discriminates glycaemic variability, while mean absolute relative difference (MARD) is the CGM industry standard. Glucose-specific RMSE (gRMSE) incorporates Clarke Error Grid-inspired penalties^33^ to emphasise clinical relevance by penalising potentially harmful therapeutic errors. Detailed metric definitions are provided in Supplementary Materials. We investigated hypoglycaemia prediction performance by converting model outputs into a binary event label using a clinically motivated criterion: an event was defined when the predicted glucose trajectory fell below 70 mg/dL within the subsequent 60 min. Model performance was assessed using standard classification metrics, including accuracy (ACC), precision (PREC), sensitivity (SEN), and specificity (SPEC). To account for the low prevalence of hypoglycaemia (Supplementary Table S2), we additionally report metrics that are less sensitive to class imbalance: area under the receiver operating characteristic curve (AUROC), area under the precision–recall curve (AUPRC), F1 score, and Matthews correlation coefficient (MCC).

In GluLLM, the only trainable components are the lightweight encoder and decoder modules, implemented as stacks of fully connected layers. We refer to this encoder–decoder pair collectively as the adaptor, as it enables task-specific input projection and output mapping while keeping the LLM backbone frozen. The adaptor has significantly fewer trainable parameters than full LLM fine-tuning or even parameter-efficient fine-tuning approaches, such as LoRA.^34^ The computational complexity of this design is comparable to that of an extreme case of LoRA applied to a single Transformer layer. However, even with this efficient design, training GluLLM with larger models such as LLaMA 8B and Mistral 7B remains challenging on an NVIDIA L4 GPU with 24 GB of RAM, even with a single batch of data, due to the significant computational demands of LLM inference. To address this, we distributed the Transformer layers of these two models across several GPUs to enable model training. For smaller LLMs, inference can be performed on a single GPU; thus, we employed standard data parallelism by distributing batches across multiple GPUs to accelerate training.

We conducted comprehensive performance comparisons between GluLLM and various existing deep learning time series forecasting approaches. The baseline methods encompass four architectural categories: Transformer-based models including Autoformer,^35^ Crossformer,^36^ FEDformer,^37^ Informer,^38^ iTransformer,^39^ non-stationary Transformer (NST),^40^ PatchTST,^41^ and vanilla Transformer; multilayer perceptron (MLP) architectures such as N-Beats,^42^ DLinear,^43^ and TiDE^44^; CNNs including TCNs^13^ and TimesNet^45^; and RNN architectures including LSTM^14^ and GRU.^15^ To enhance the generalisation capabilities of non-LLM models, we applied a model-agnostic meta-learning framework for domain generalisation, which has effectively mitigated the high inter-individual variability in our recent work.^16^

### Smartphone platform implementation

We implemented GluLLM on iOS smartphones, as on-device LLMs will be available on next-generation iPhones. For development, we utilised Swift 5 and Xcode 16.2 as the integrated development environment. It should be noted that we focused solely on evaluating the memory and CPU usage of the proposed LLM-driven health application rather than developing a complete user interface. LLM inference was performed using the llama.cpp library.^46^ For integration, the GluLLM encoder and decoder components were converted to Core ML format to facilitate efficient interaction with the LLaMA model. Real-time CPU and memory usage were assessed using Xcode's profiling tools. The evaluation was conducted on an iPhone 14 Pro Max, equipped with an Apple A16 Bionic chip, a 6-core CPU, and 6 GB of RAM, as well as on an iPhone SE (2020), featuring an A13 Bionic chip, a 6-core CPU, and 3 GB of RAM. We initially deployed LLaMA 3.2 1B in GGUF format using full 16-bit weights without quantisation; however, to enable stable real-time inference on an iPhone SE, we subsequently applied 5-bit quantisation using the Hugging Face Transformers library (v4.44.0), and report the results using this quantised configuration.

### Ethics

For REPLACE-BG, the original randomised clinical trial received ethics approval from the institutional review boards at participating US sites (ClinicalTrials.gov NCT02258373), and written informed consent was obtained from all participants. For the Móstoles cohort, the original observational study was approved by the Ethics Committee of University Hospital of Móstoles (Madrid, Spain), and written informed consent was obtained from all participants. As our work is a secondary analysis of fully de-identified, publicly available datasets, no additional ethics approval was required for the present analyses.

### Statistics

This study is a retrospective analysis of previously collected datasets for model development and validation. No formal sample-size calculation was performed because sample size was determined by the available cohorts. Data were split using a participant-disjoint strategy, and external validation was performed on an independent cohort (Móstoles) without re-training. Performance metrics are reported as mean ± SD. For comparisons between GluLLM and baseline methods, we used paired statistical testing on matched evaluation units under the same split and reported two-sided p-values; *p* < 0.05 was considered statistically significant. Hypoglycaemia events were defined using a prespecified rule (predicted glucose < 70 mg/dL within 60 min), and we report AUROC and AUPRC alongside F1 score and MCC to support evaluation under class imbalance.

### Role of funders

The funders had no role in the study design, data collection, data analysis, interpretation of the results, or writing of this manuscript.

## Results

### Zero-shot glucose prediction performance

Our initial experiment evaluated the zero-shot learning performance of LLMs for glucose prediction, which refers to the ability of a pretrained model to perform a specialised task without any additional fine-tuning or training. We constructed the prompts that included comprehensive demographic information (age, sex, BMI, and HbA1c) alongside 6 h of historical CGM readings. These structured prompts instructed the model to generate glucose predictions in a standardised format, allowing us to assess the baseline performance of pretrained LLMs. The detailed prompt template is provided in the Supplementary Materials.

To ensure practical deployment of digital health systems, we selected compact open-source models from leading LLM families, including LLaMA (Meta), Mistral (Mistral AI), Gemma (Google), Phi (Microsoft), OpenELM (Apple), and GPT (OpenAI). Table 1 presents detailed information on model versions, parameter sizes, and response characteristics.

**Table 1:** Zero-shot glucose prediction performance of pretrained lightweight LLMs.

Model	Size	Response quality
LLaMA 3.2	1B	Attempted a response but misinterpreted the task, referring to blood pressure and concluding without a specific prediction.
LLaMA 3.2	3B	Initiated a structured statistical approach but contained calculation inaccuracies and did not produce a concrete prediction.
LLaMA 3.1	8B	Offered theoretical time series methodology discussion in multiple steps but avoided calculations, providing only a placeholder “0” as the final answer.
Mistral 0.2	7B	Started explaining analytical forecasting methods like linear regression in Python code but did not deliver any predictions.
Gemma 2	2B	Emphasised general medical considerations and disclaimers without directly addressing the prediction component of the task.
Phi-3.5 mini	3.8B	Discussed machine learning strategies (e.g., RNNs with PyTorch) but the response lacked focus and did not complete the prediction.
OpenELM	270M	Returned a series of other clinical features (e.g., blood pressure) but did not address the glucose prediction objective.
GPT-2	1.5B	Generated off-topic text discussing unrelated medical studies with no attempt at glucose prediction.

Size denotes the number of model parameters; B denotes billions of parameters; M denotes millions of parameters.

Our evaluation revealed a significant performance gap: none of these LLMs demonstrated the capability to produce clinically viable glucose predictions. Rather than generating the requested numerical predictions, most models produced theoretical explanations of time series prediction methodologies (e.g., linear regression and RNNs) accompanied by Python implementations. These responses represent a substantial deviation from the required clinical functionality, requiring technical expertise beyond that possessed by most users and healthcare providers, and demonstrate a fundamental misalignment between the models’ generative tendencies and the specific requirements of prediction tasks. These findings highlight the critical limitations of applying general-purpose LLMs directly to specialised clinical tasks without appropriate adaptations.

### Enhancing glucose prediction in pretrained LLMs with GluLLM

To address the performance limitations of pretrained LLMs in glucose prediction, we propose the GluLLM framework (Fig. 1), which enhances predictive accuracy through targeted adaptation. Our results demonstrate that when augmented with GluLLM, all evaluated LLMs successfully provided viable glucose predictions.

**Fig. 2:**
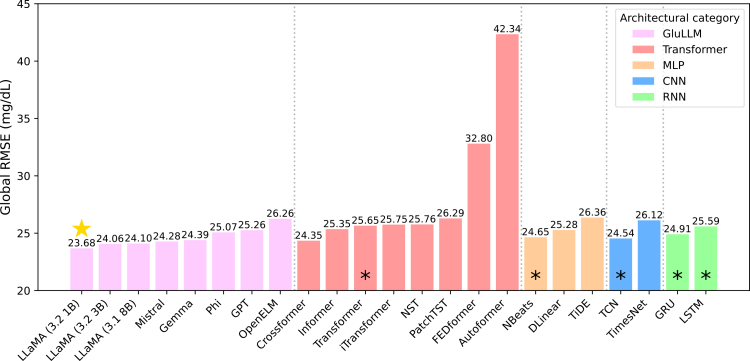
Global RMSE for glucose time series across different model architectures with a 60-min prediction horizon, evaluated on the held-out REPLACE-BG test set (n = 46 participants). The colour coding represents distinct architectural categories: violet for LLM-based models, light red for Transformer-based models, light orange for MLP-based models, blue for CNN-based models, and green for RNN-based architectures. Black asterisk markers indicate models previously explored in glucose prediction literature, while the gold star highlights the best-performing model in our evaluation.

Fig. 2 presents the global RMSE values comparing predicted sequences with actual CGM sequences. The results demonstrate that LLaMA 3.2 models achieved superior performance, with the 1B version ranking at the top. Other LLMs in our evaluation exhibited performance comparable to the average level of deep learning baseline methods. Among the tested LLMs, OpenELM delivered the lowest performance, which may be attributed to its significantly smaller parameter count compared to other architectures.

Following the global RMSE evaluation, we selected the top-performing model from each architecture family for detailed comparison. Table 2 presents the results of four regression metrics at 30- and 60-min prediction horizons, following standard evaluation settings in glucose prediction tasks. Specifically, we report RMSE, MAE, MARD, and gRMSE. Lower values across all metrics indicate better predictive performance. Among the evaluated LLMs, we selected the LLaMA 3.2 1B model as the core architecture of GluLLM for all subsequent experiments due to its strong performance and efficient resource usage. GluLLM achieved the best performance at both prediction horizons across all regression metrics when compared with the top models from each deep learning family, with statistically significant improvements over the best baseline Crossformer (*p* < 0.001 for all metrics on both datasets). Furthermore, when evaluated on the external Móstoles dataset, GluLLM consistently ranked first across all metrics and both time horizons, demonstrating strong generalisability and robustness to different populations and clinical contexts.

**Table 2:** Regression metrics for glucose prediction (mean ± SD) on the held-out REPLACE-BG test set and external Móstoles cohort at 30-min and 60-min horizons.

Method	30 min				60 min			
	RMSE	MAE	MARD (%)	gRMSE	RMSE	MAE	MARD (%)	gRMSE
REPLACE-BG								
GluLLM	**20.6** ± **3.5**	**14.7** ± **2.6**	**10.6** ± **2.4**	**25.6** ± **4.8**	**35.8** ± **6.0**	**26.6** ± **4.5**	**19.6** ± **4.5**	**46.5** ± **8.5**
Crossformer	21.1 ± 3.6	15.0 ± 2.6	10.6 ± 2.4	26.2 ± 4.9	36.3 ± 6.0	26.9 ± 4.5	19.6 ± 4.4	47.2 ± 8.5
NBeats	21.4 ± 3.7	15.3 ± 2.7	10.9 ± 2.5	26.6 ± 5.0	36.6 ± 6.1	27.3 ± 4.6	19.9 ± 4.5	47.6 ± 8.6
TCN	21.3 ± 3.7	15.2 ± 2.7	10.8 ± 2.4	26.7 ± 5.0	36.4 ± 6.1	27.2 ± 4.5	19.7 ± 4.4	47.7 ± 8.6
GRU	22.1 ± 3.7	15.6 ± 2.6	10.9 ± 2.3	26.9 ± 4.9	38.1 ± 6.3	28.0 ± 4.7	19.8 ± 4.0	48.4 ± 8.8
Móstoles								
GluLLM	**9.6** ± **2.9**	**6.9** ± **2.1**	**6.6** ± **1.9**	**10.3** ± **3.3**	**16.0** ± **5.5**	**11.7** ± **4.0**	**11.0** ± **3.3**	**17.6** ± **6.7**
Crossformer	10.1 ± 2.9	7.5 ± 2.0	7.4 ± 2.0	11.0 ± 3.4	17.9 ± 4.6	14.0 ± 3.4	14.0 ± 3.8	20.4 ± 6.2
NBeats	10.1 ± 3.0	7.4 ± 2.2	7.2 ± 2.1	11.1 ± 3.5	18.1 ± 4.7	14.1 ± 3.5	14.2 ± 4.1	20.7 ± 6.4
TCN	10.2 ± 3.0	7.6 ± 2.1	7.4 ± 2.2	11.2 ± 3.5	18.5 ± 4.6	14.7 ± 3.4	14.9 ± 4.3	21.3 ± 6.5
GRU	10.8 ± 3.2	8.1 ± 2.3	8.0 ± 2.5	12.1 ± 4.0	18.8 ± 4.1	15.3 ± 3.0	15.7 ± 3.9	21.7 ± 5.9

RMSE, root mean square error; MAE, mean absolute error; MARD, mean absolute relative difference; gRMSE, glucose-specific root mean square error.

Lower values indicate better performance for all metrics. Bold values indicate the best-performing model for each metric.

Further evaluation was conducted to assess whether the glucose predictions could effectively indicate adverse glycaemic events, particularly hypoglycaemia, which can lead to severe complications in a short period of time. In this analysis, we used classification metrics to determine whether the predicted glucose time series could accurately detect hypoglycaemic events, defined as glucose levels below 70 mg/dL. For the reported metrics, higher values uniformly indicate better predictive performance. We chose to report overall performance across all testing data rather than individual-level results because some participants experienced no hypoglycaemic events during the monitoring period, making certain classification metrics incalculable for these individuals.

In Table 3, GluLLM achieved the highest scores for AUROC, AUPRC, F1, and MCC, indicating the best overall classification performance among all models. Notably, GluLLM also significantly improved sensitivity, detecting a greater proportion of hypoglycaemic events on both datasets: an improvement of 11.2% on the REPLACE-BG dataset and 10.2% on the Móstoles dataset compared with the second-best models, Crossformer and GRU, respectively. These gains suggest that implementing GluLLM could potentially lead to a significant reduction in the occurrence of hypoglycaemic events.

**Table 3:** Hypoglycaemia prediction performance on the held-out REPLACE-BG test set and external Móstoles cohort.

Method	ACC	PREC	SEN	SPEC	AUROC	AUPRC	F1	MCC
REPLACE-BG								
GluLLM	**95.1**	85.5	**59.6**	98.9	**0.793**	**0.551**	**0.703**	**0.690**
Crossformer	94.9	91.1	53.6	99.4	0.765	0.534	0.675	0.676
NBeats	95.0	92.1	53.4	99.5	0.765	0.538	0.676	0.680
TCN	94.8	**94.3**	50.4	**99.7**	0.751	0.524	0.657	0.669
GRU	95.0	93.1	52.4	99.6	0.760	0.535	0.671	0.677
Móstoles								
GluLLM	**97.9**	85.4	**68.9**	99.4	**0.841**	**0.604**	**0.763**	**0.756**
Crossformer	97.9	96.9	59.0	**99.9**	0.794	0.592	0.733	0.747
NBeats	97.9	93.8	62.3	99.8	0.811	0.600	0.749	0.755
TCN	97.7	**99.3**	54.7	99.7	0.773	0.565	0.705	0.728
GRU	97.6	77.4	62.5	99.4	0.766	0.580	0.709	0.720

Events were defined using a decision rule of predicted glucose <70 mg/dL within a 60-min horizon. ACC, accuracy; PREC, precision; SEN, sensitivity; SPEC, specificity; AUROC, area under the receiver operating characteristic curve; AUPRC, area under the precision–recall curve; F1, F1 score; MCC, Matthews correlation coefficient.

Higher values indicate better performance for all metrics. Bold values indicate the best-performing model for each metric.

The performance gains achieved by GluLLM can be attributed to several key design elements. First, incorporating contextual information significantly enhances LLM performance in time series forecasting. In our case, demographic characteristics closely related to an individual's metabolic status, such as age, sex, BMI, and HbA1c, substantially influence glucose dynamics. Integrating this information enables the personalisation of a population-level model, allowing it to generalise effectively to unseen individuals without the need for fine-tuning or post-training, as demonstrated in our ablation study on the Móstoles dataset (Supplementary Fig. S3). Second, the autoregressive architecture and next-token prediction allow GluLLM to translate the token transitions learnt during pretraining into meaningful glucose trajectories over time, leveraging the core strengths of language models. This flexibility also enables extending prediction horizons. For 2-h predictions, we append previously predicted glucose tokens and perform additional next-token prediction rounds. Preliminary results demonstrate over 90% clinically acceptable predictions across both datasets (Supplementary Fig. S4). Finally, the TDI module enables real-time adaptation by integrating textual inputs describing daily activities. This allows the model to account for transient glucose fluctuations, such as those resulting from insulin interventions (Supplementary Fig. S3). Notably, the incremental benefit of the TDI module was smaller in the Móstoles cohort than in REPLACE-BG. This difference is attributable to the available event inputs: in REPLACE-BG (type 1 diabetes), TDI incorporates insulin delivery information, a major time-dependent determinant of short-term glucose dynamics under intensive management, whereas comparable insulin event data are not available in the Móstoles dataset, thereby constraining the contribution of TDI. This ablation study also includes a sensitivity analysis using forward-fill imputation (Supplementary Fig. S3); given the low proportion of missing CGM values (mean 2.7% in REPLACE-BG and 0.7% in Móstoles), the imputation strategy had no material impact on performance.

### Memory and CPU footprint of GluLLM on smartphone platforms

**Fig. 3:**
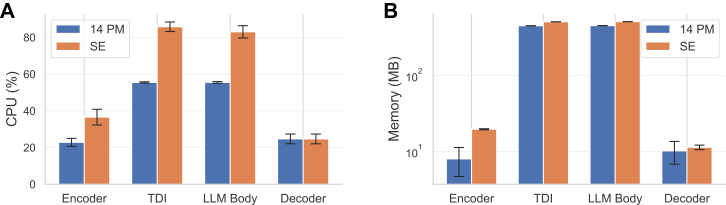
Peak CPU and memory usage of GluLLM across smartphone platforms (n = 10 independent runs). Blue and orange bars represent the performance of the iPhone 14 Pro Max (14 PM) and iPhone SE 2020 (SE), respectively. Error bars indicate the mean ± standard deviation. **A.** Peak CPU usage by component **B.** Peak memory usage by component.

To demonstrate feasibility, we implemented GluLLM on two smartphone devices: the iPhone 14 Pro Max (representing a mainstream high-end device) and the iPhone SE 2020 (representing a resource-constrained platform). Fig. 3 shows the peak CPU and memory usage during GluLLM inference on both devices. Peak values are reported because sudden computational spikes can exceed device capabilities and risk freezing or crashing the system. The iPhone 14 Pro Max consumed a maximum of 55.5% CPU and 453.9 MB of memory (7.4% of the device's total 6 GB memory capacity), while the iPhone SE 2020 consumed a maximum of 85.8% CPU and 512.2 MB of memory (16.7% of the device's total 3 GB memory capacity). When examining the computational demands of individual components, we found that the encoder and decoder modules required substantially fewer resources compared with the TDI module and LLM body, consuming a maximum of 24.7% CPU and 10.2 MB of memory on the iPhone 14 Pro Max, and 36.6% CPU and 19.7 MB of memory on the iPhone SE 2020.

The average memory consumption for running GluLLM was 139.1 MB (2.5%) on the iPhone 14 Pro Max and 178.1 MB (5.8%) on the iPhone SE, both exhibiting low energy impact. On the iPhone 14 Pro Max, the mean warm-up time (model initialisation and loading) was 2.726 s, with steady-state latencies of 0.180 s for the LLM backbone and 1.061 s for the TDI. On the iPhone SE, the mean warm-up time was 40.599 s, with steady-state latencies of 1.066 s (LLM backbone) and 6.222 s (TDI). The warm-up cost primarily reflects one-off layer loading into CPU memory and is incurred once per app session; in contrast, the steady-state latency represents the per-prediction runtime. Notably, steady-state inference on both devices remains substantially shorter than the typical CGM sampling interval (5 min), supporting the feasibility of real-time decision support in day-to-day use.

## Discussion

In this work, we proposed GluLLM, a deep learning framework designed for two glucose management tasks: multi-horizon glucose trajectory prediction (regression) and hypoglycaemia prediction (classification). GluLLM uses the preceding 6 h of CGM time series as its primary input via a dedicated wearable encoder, incorporates demographic and clinical variables (age, sex, BMI, and HbA1c) through a structured, text-based EHR prompt, and introduces a TDI module to encode real-time events, including recent insulin delivery. We trained GluLLM using data from 180 participants in REPLACE-BG (26 weeks; ∼9.43 million CGM measurements) and evaluated performance on 46 held-out REPLACE-BG participants, with external validation in 207 participants from the Móstoles cohort (24–72 h of CGM monitoring). Compared with 15 established deep learning baselines, GluLLM achieved superior performance, including a 30-min RMSE of 20.6 ± 3.5 mg/dL on 46 hold-out REPLACE-BG participants and improved hypoglycaemia prediction (AUROC 0.79; AUPRC 0.55), and it maintained strong performance under external validation in 207 participants in Móstoles (RMSE 9.6 ± 2.9 mg/dL; AUROC 0.84; AUPRC 0.60). Ablation analyses indicate that both the EHR prompt and TDI module contribute meaningfully to predictive performance. Subject to appropriate regulatory approvals and prospective evaluation, it could be integrated into existing CGM smartphone workflows as a companion module that provides multi-horizon glucose predictions and hypoglycaemia warnings alongside standard CGM displays. Future studies should assess safety, usability, alert burden, and workflow integration, with ongoing monitoring for dataset shift and periodic re-validation across devices and populations before any clinical implementation.

In GluLLM, the LLM backbone remains a standard decoder-only language model and therefore retains its pretrained parameters; in this work, we use task-specific input projection and output heads to map between multimodal health inputs and the model's latent representation, and to decode the hidden states into a subsequent glucose trajectory. Thus, the framework does not sacrifice text generation but rather repurposes the output interface for forecasting in the present experiments. Recent work has also questioned whether LLM backbones confer consistent benefits for generic time-series forecasting benchmarks^47^; however, those evaluations largely focus on comparatively homogeneous datasets (e.g., electricity or weather) and do not capture the complexity of clinical decision support, where outcomes are shaped by time-stamped events (e.g., insulin delivery) and individual-level clinical context (e.g., BMI). In our design, we also adopt an autoregressive, next-token prediction formulation following AutoTimes.^22^ In their ablation analyses, the authors report that replacing the LLM backbone with smaller alternatives leads to reduced performance, suggesting that the backbone can contribute to modelling long-range dependencies and interactions between heterogeneous inputs in this setting. We selected an LLM backbone because it provides (i) a flexible mechanism to incorporate clinical context via structured EHR prompts, supporting personalised adaptation without subject-specific fine-tuning, and (ii) a shared on-device backbone that can be reused across tasks by swapping lightweight adaptors, aligning with emerging smartphone deployments of local LLMs. The framework also supports additional wearable modalities via dedicated adaptors (e.g., combining wrist-worn sensors with CGM^15^) and is readily extensible to other conditions.

Nevertheless, LLM-driven approaches may appear computationally intensive for traditional forecasting, but their value is more apparent in biomedical settings where predictions depend on heterogeneous inputs, including time-series signals and clinically meaningful context. In our implementation, the LLM backbone is frozen throughout training, and learning is confined to the lightweight encoder–decoder adaptor modules implemented as fully connected neural networks. As a result, parameter updates and optimiser state are restricted to these small modules, substantially reducing trainable parameters and training overhead compared with full-model fine-tuning or LoRA-style adaptation.^34^ We chose such adaptors over LoRA because our primary goal is to improve the interpretation of wearable data rather than to improve natural language generation. Input representation quality is more critical than fluent textual outputs in our context. Future work will explore integrating LoRA for applications requiring response generation, such as diagnosis suggestions or treatment recommendations, which would be evaluated with clinicians for real-world utility. Furthermore, generating explanatory text alongside the predictions may provide insight into the reasoning of the model,^48^ representing an important direction for interpretation research.

Beyond training efficiency, we demonstrated a feasible deployment of GluLLM on resource-constrained smartphones. On-device deployment preserves the privacy of sensitive health data and could support health management without service interruptions. Steady-state inference latency was well within the CGM sampling interval, but the initial model-loading time on lower-resource smartphone hardware was more noticeable and may affect user experience; reducing warm-up latency through improved caching, model loading, and device-specific optimisation remains an important area for future work. Although the LLM body and TDI are primary contributors to CPU, memory, and power consumption, feeding TDI-processed contextual information directly into the LLM would substantially increase computational demands due to the quadratic complexity of the Transformer layers with input length. By separating daily contextual information into the TDI module, we preserve modelling flexibility while improving training and inference efficiency. Furthermore, TDI embeddings often remain unchanged for days, particularly when no recent insulin administration occurs, enabling precomputation and storage to further reduce computational overhead. Collectively, these design choices support the feasibility of deploying GluLLM on smartphones. The lightweight encoder and decoder modules could support multiple task-specific adaptors within a shared on-device framework, although this was not evaluated in the present study. In future work, knowledge distillation^49^ and parameter quantisation may further improve deployability by reducing memory footprint and latency, enabling use on more resource-constrained devices and facilitating larger-capacity models under limited hardware budgets.

However, several limitations still exist in this study. First, the REPLACE-BG cohort was predominantly White non-Hispanic (91.6%), and ethnicity was not reported for the Móstoles cohort, limiting assessment of performance across demographic strata. In addition, although our sample sizes support benchmarking across two clinical datasets, they may be insufficient to draw robust conclusions for under-represented subgroups. Future work should prioritise prospective evaluation in larger, more diverse cohorts and assess performance stratified by demographic and clinical characteristics. Second, our on-device evaluation focused on CPU and memory footprint and inference latency on two smartphone models; real-world performance will depend on background device load, operating system constraints, and future hardware and software optimisations. Finally, this study primarily evaluates predictive performance rather than clinical impact. Prospective studies are therefore needed to assess clinical benefit, alert burden, user adherence, and integration into routine care workflows.

## Contributors

The study was conceptualised by TZ and AN-H. TZ developed the methodology, implemented the model, curated the data, performed the analyses, and generated the visualisations. TZ and AN-H jointly accessed and verified all underlying data. Project supervision, access to computational resources, and funding acquisition were overseen by JH and AN-H. TZ drafted the initial manuscript, and JH and AN-H provided critical review and revision. All authors approved the final manuscript and accept responsibility for the decision to submit for publication.

## Data sharing statement

Datasets used in this study are publicly available. The REPLACE-BG dataset was obtained from Aleppo et al. (2017), “REPLACE-BG RCT Protocol 10-27-15 v2.0,” available at https://public.jaeb.org/dataset/546. The analyses, interpretations, and conclusions presented herein are solely the responsibility of the authors and have not been reviewed or endorsed by Aleppo et al. The Móstoles dataset was obtained from Colás et al. (2019), available at https://journals.plos.org/plosone/article?id=10.1371/journal.pone.0225817. The code implementation of GluLLM is available on GitHub (https://github.com/tndrg/GluLLM).

## Declaration of interests

J.H. is an employee of Novo Nordisk Research Centre Oxford and also a shareholder of Novo Nordisk. T.Z. received salary support through a Novo Nordisk Postdoctoral Fellowship run in partnership with the University of Oxford, which also supported research-related travel and conference/meeting attendance. A.N.-H. declares no competing interests.
