# Supplementary material for: Empowering digital health management with on-device large language models for glucose prediction: a model development and validation study
Source: eBioMedicine. 2026 Jun 25;129:106343. doi: 10.1016/j.ebiom.2026.106343 (PMC13320499; doi:10.1016/j.ebiom.2026.106343)
Supplement: Supplementary Material [file mmc1.docx]

**Empowering digital health management with on-device large language models for glucose prediction: a model development and validation study**

Taiyu Zhu^1,2^, Joanna Howson^3^, and Alejo Nevado-Holgado^1^

^1^ *Department of Psychiatry, University of Oxford, Warneford Hospital, Oxford OX3 7JX, UK*

^2^ *Department of Biostatistics & Health Informatics, Institute of Psychiatry, Psychology & Neuroscience, King’s College London, 16 De Crespigny Park, London SE5 8AF, UK*

^3^ *Novo Nordisk Research Centre Oxford, Old Road Campus, Roosevelt Drive, Oxford OX3 7FZ, UK*

# Supplementary materials

## Regression metric definitions

Three widely used regression metrics for glucose prediction were employed: root mean square error (RMSE), mean absolute error (MAE), and mean absolute relative difference (MARD), defined as:

$$RMSE=\sqrt{\frac{1}{L_{T}}\sum_{t=1}^{L_{T}} \left( G_{t}-Ĝ_{t} \right)^{2}}$$

$$MAE=\frac{1}{L_{T}}\sum_{t=1}^{L_{T}} |G_{t}-Ĝ_{t}|$$

$MARD=\frac{1}{L_{T}}\sum_{t=1}^{L_{T}} \frac{|G_{t}-Ĝ_{t}|}{G_{t}}\times100\%$ (S1)

where *L_T_* denotes the total number of testing samples, *G_t_* is the ground truth blood glucose measurement at time *t*, and *Ĝ_t_* is the corresponding predicted value.

To account for glucose-specific prediction error, we further used glucose-specific RMSE (gRMSE) [1] as follows:

$gRMSE=\sqrt{\frac{1}{L_{T}}\sum_{t=1}^{L_{T}} P\left( G_{t},Ĝ_{t} \right)\left( G_{t}-Ĝ_{t} \right)^{2}}$ (S2)

$$P\left( G_{t},Ĝ_{t} \right)=1+\alpha_{L}{\sigmā}_{G_{t}\leq T_{L},\beta_{L}}\left( G_{t} \right)\sigma_{Ĝ_{t}\geq G_{t},\gamma_{L}}\left( Ĝ_{t},G_{t} \right)$$

$+\alpha_{H}\sigma_{G_{t}\geq T_{H},\beta_{H}}\left( G_{t} \right){\sigmā}_{Ĝ_{t}\leq G_{t},\gamma_{H}}\left( Ĝ_{t},G_{t} \right)$ (S3)

where *P* is a penalty function inspired by the Clarke Error Grid (CEG), designed to penalise overestimation in hypoglycaemia and underestimation in hyperglycaemia. Definitions of the sigmoid-like functions σ̄, σ, and the corresponding parameters [*α_L_*, *β_L_*, *γ_L_*, *T_L_*, *α_H_*, *β_H_*, *γ_H_*, *T_H_*] can be found in the literature [1].

## Zero-shot prompt template

This section provides the zero-shot prompt template used to evaluate LLM zero-shot glucose prediction performance.

*“A [age]-year-old [sex] individual has an HbA1c of [HbA1c]% and a BMI of [BMI]. Based on their continuous glucose monitoring (CGM) data recorded every 5 minutes over the past 6 hours [historical_readings], predict their glucose levels for the next hour at 5-minute intervals.”*

# Supplementary figures

## Fig. S1. Encoder–decoder adapter architecture in GluLLM

Supplementary Fig. S1 depicts the architecture of the encoder and decoder modules that constitute the GluLLM adapter.


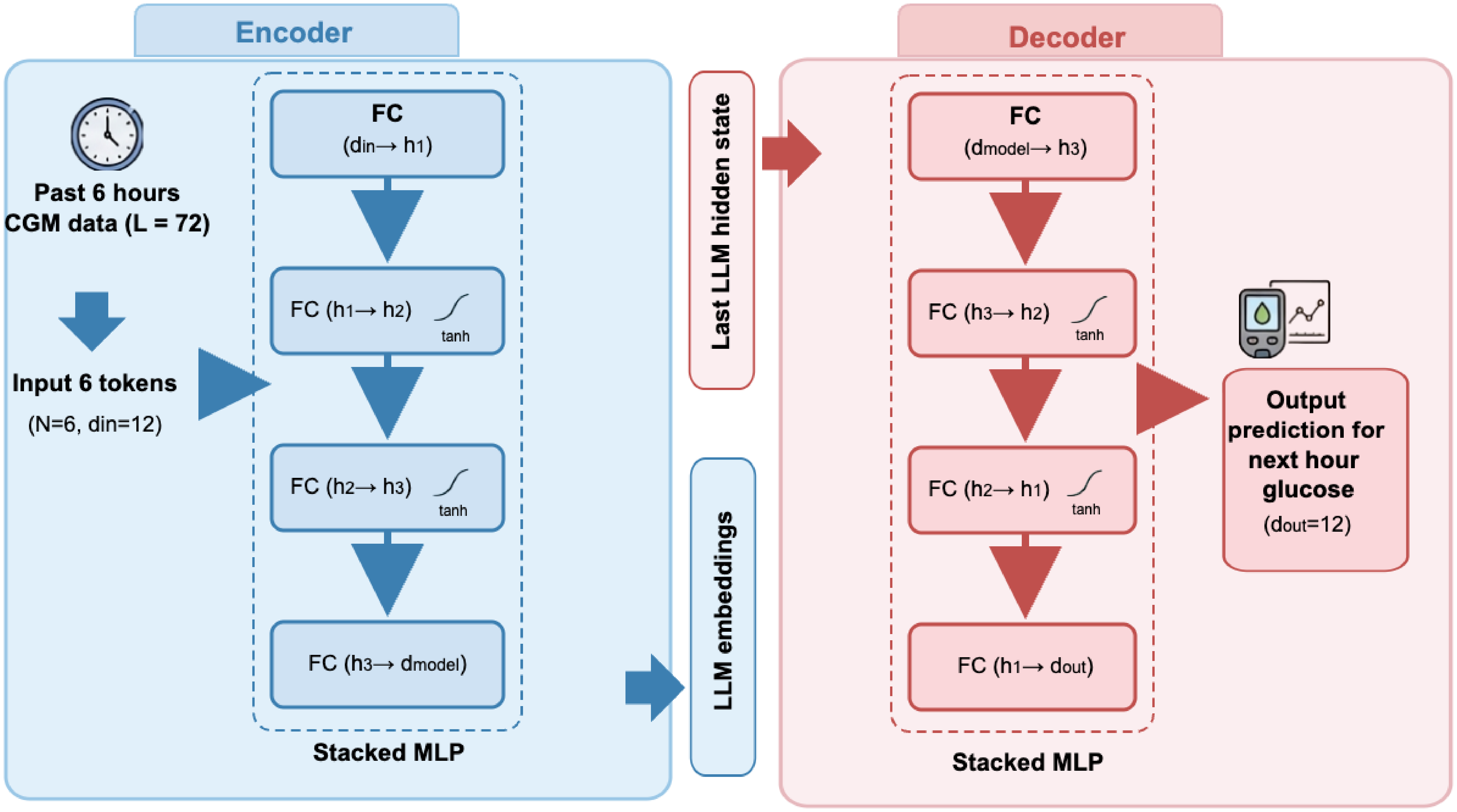


**Supplementary Fig. S1:** Schematic of the GluLLM adapter. The encoder and decoder comprise stacks of fully connected multilayer perceptron (MLP) layers that project inputs into, and outputs from, the LLM embedding space.

## Fig. S2. Cohort stratification and cross-validation framework

Supplementary Fig. S2 illustrates the cohort stratification used in the five-fold cross-validation framework, as well as the procedures for model training and hold-out testing.


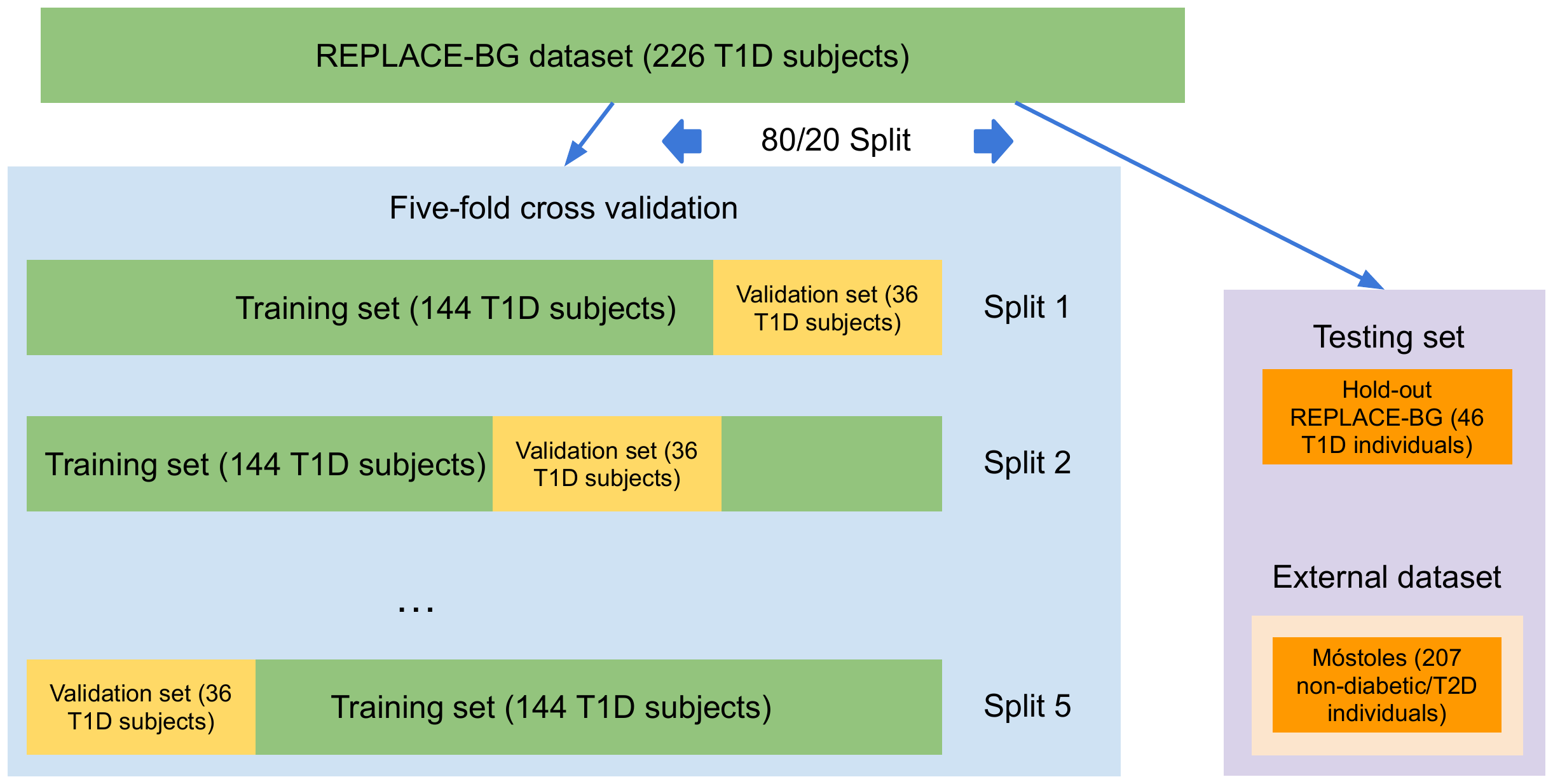


**Supplementary Fig. S2:** Data partitioning strategy. The REPLACE-BG dataset was first divided into a development set, which was used for training and validation in five-fold cross-validation, and a hold-out testing set, with splitting performed on an individual basis to prevent data leakage. The Móstoles dataset served as an independent external validation cohort to assess generalisation performance.

## Fig. S3. Ablation and imputation sensitivity analysis

Supplementary Fig. S3 shows the ablation and imputation sensitivity analyses for GluLLM and reports a sensitivity analysis for missing-data handling using a simple forward-fill strategy (last observation carried forward).


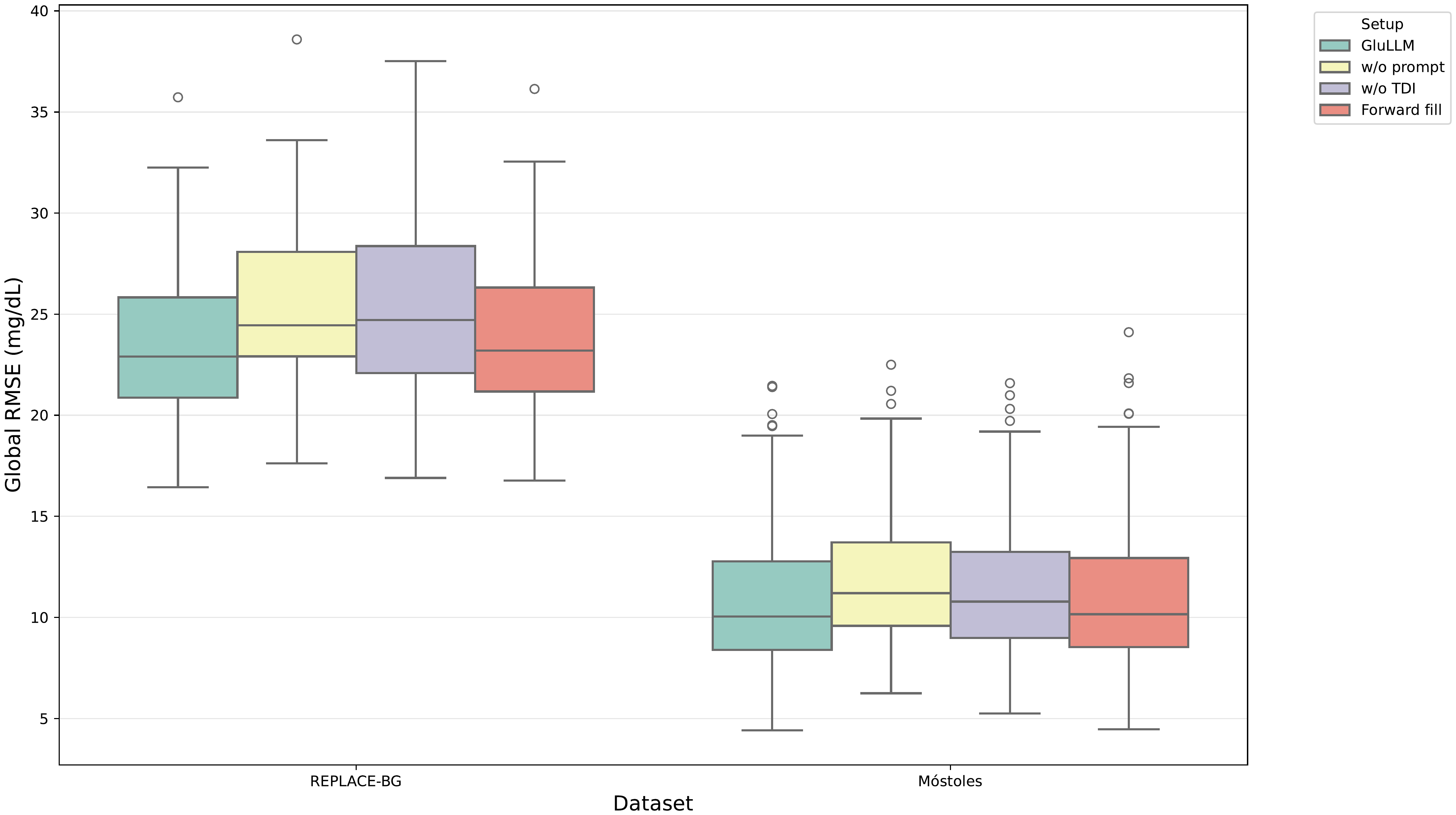


**Supplementary Fig. S3:** Ablation study evaluating the contributions of the EHR prompt, TDI module, and missing-data imputation strategy to glucose prediction performance. Global RMSE over a 60-minute prediction horizon is used as the evaluation metric. Each dot represents the RMSE for an individual participant in the respective dataset. In the box plots, the lower and upper hinges correspond to the first (Q1) and third (Q3) quartiles, respectively; the central line indicates the median; and the whiskers extend to 1.5 times the interquartile range (IQR). Circles denote statistical outliers.

## Fig. S4. Clarke Error Grid analysis at a two-hour prediction horizon

Supplementary Fig. S4 shows the Clarke Error Grid (CEG) analysis at a two-hour prediction horizon. The proportion of predictions falling within CEG zones A+B was high in both datasets, with 92.26% in REPLACE-BG and 99.09% in Móstoles.


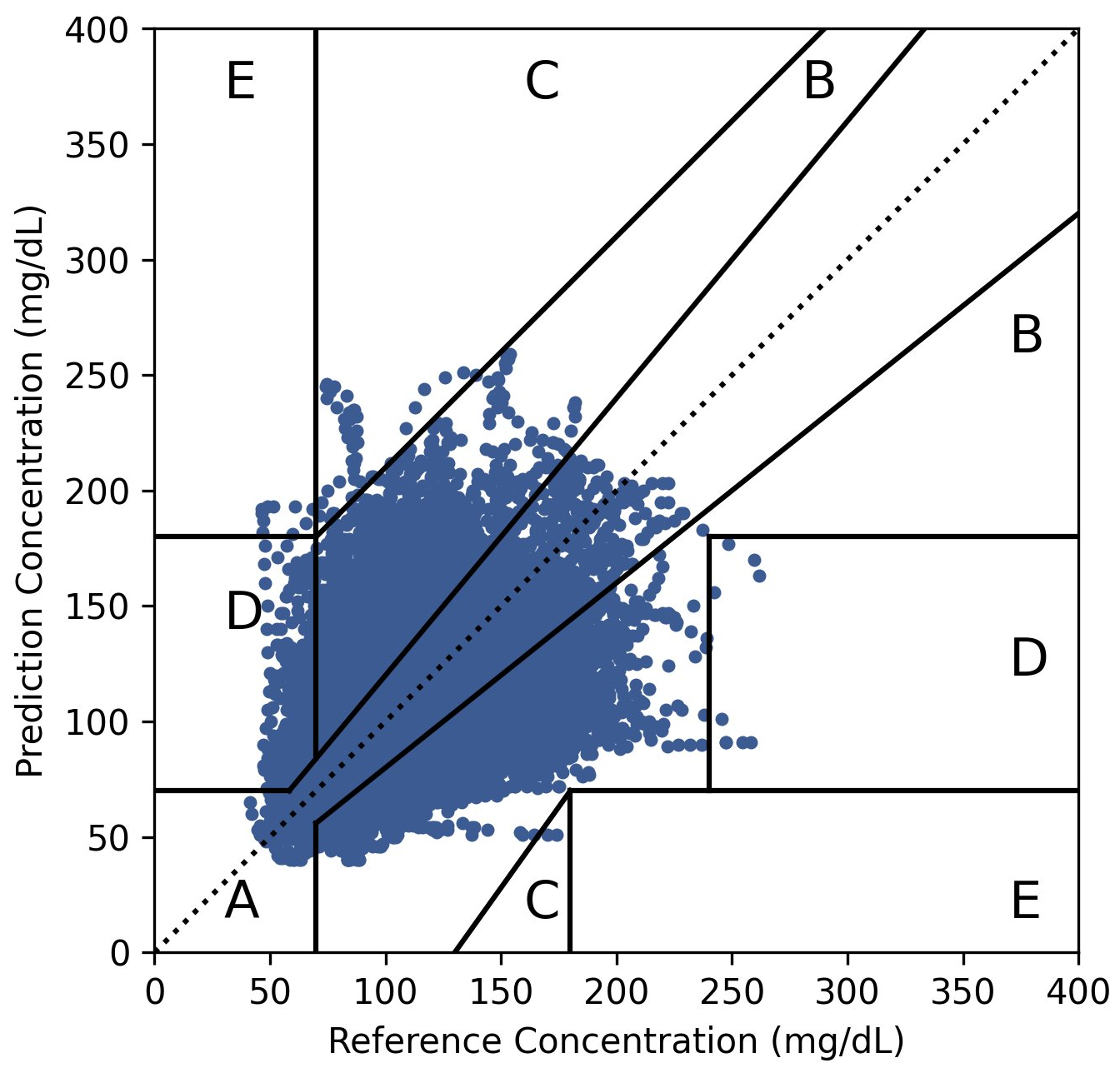


**Supplementary Fig. S4:** Clarke Error Grid (CEG) analysis comparing predicted versus observed glucose values at a two-hour prediction horizon. Points are partitioned into zones (A–E) according to the potential clinical relevance of prediction errors; zones A and B indicate agreement generally considered acceptable under the CEG framework, whereas zones C–E indicate increasing potential for clinically relevant error.

# Supplementary tables

Supplementary Tables S1 and S2 summarise participant demographics, study characteristics, and CGM-derived glycaemic measures for the REPLACE-BG and Móstoles cohorts. Supplementary Table S3 summarises the hyperparameters used in this work.

**Supplementary Table S1:** Participant demographics and study characteristics.

| **Characteristic** | **REPLACE-BG** | **Móstoles** |
| --- | --- | --- |
| **Study design** | | |
| Recruitment setting | Multi-centre clinical trial (14 US sites) | Single Spanish outpatient clinic |
| **Sample size** | | |
| Total participants, n | 226 | 207 |
| Male, n (%) | 114 (50.4) | 103 (49.8) |
| Female, n (%) | 112 (49.6) | 104 (50.2) |
| **Race/ethnicity** | | |
| White, non-Hispanic, n (%) | 207 (91.6) | Not reported |
| Hispanic/Latino, n (%) | 9 (4.0) | Not reported |
| Black/African American, n (%) | 5 (2.2) | Not reported |
| Asian, n (%) | 4 (1.8) | Not reported |
| Other/unknown, n (%) | 1 (0.4) | Not reported |

**Supplementary Table S2:** CGM-derived glycaemic measures overall and by sex. Values are mean ± SD unless otherwise stated.

| **Measure** | **REPLACE-BG** | **Móstoles** |
| --- | --- | --- |
| **Glycaemic control** | | |
| Mean glucose (mg/dL), all | 159.9 ± 26.4 | 102.2 ± 12.1 |
| Male | 159.7 ± 27.6 | 103.9 ± 12.1 |
| Female | 160.0 ± 25.2 | 100.6 ± 12.0 |
| GMI (%), all | 7.1 ± 0.6 | 5.7 ± 0.3 |
| Male | 7.1 ± 0.7 | 5.8 ± 0.3 |
| Female | 7.1 ± 0.6 | 5.7 ± 0.3 |
| eA1c (%), all | 7.2 ± 0.9 | 5.2 ± 0.4 |
| Male | 7.2 ± 1.0 | 5.2 ± 0.4 |
| Female | 7.2 ± 0.9 | 5.1 ± 0.4 |
| **Time in ranges** | | |
| TBR Level 2 (%, < 54 mg/dL), all | 1.5 ± 2.3 | 0.6 ± 2.4 |
| Male | 1.6 ± 2.6 | 0.3 ± 1.3 |
| Female | 1.3 ± 2.0 | 0.9 ± 3.2 |
| TBR Level 1 (%, 54–69 mg/dL), all | 3.6 ± 3.4 | 3.2 ± 6.1 |
| Male | 3.6 ± 3.7 | 2.3 ± 4.2 |
| Female | 3.5 ± 3.0 | 4.1 ± 7.5 |
| TIR (%, 70–180 mg/dL), all | 62.5 ± 16.0 | 95.6 ± 7.7 |
| Male | 62.3 ± 16.7 | 96.7 ± 5.6 |
| Female | 62.6 ± 15.3 | 94.6 ± 9.5 |
| TAR Level 1 (%, 181–250 mg/dL), all | 22.5 ± 10.0 | 0.5 ± 2.4 |
| Male | 22.4 ± 10.6 | 0.7 ± 3.0 |
| Female | 22.5 ± 9.5 | 0.4 ± 1.6 |
| TAR Level 2 (%, > 250 mg/dL), all | 10.1 ± 9.9 | 0.0 ± 0.1 |
| Male | 10.2 ± 10.4 | 0.0 ± 0.1 |
| Female | 10.0 ± 9.4 | 0.0 ± 0.0 |
| **Glycaemic variability** | | |
| LBGI, all | 1.3 ± 1.1 | 1.7 ± 1.5 |
| Male | 1.3 ± 1.2 | 1.4 ± 1.2 |
| Female | 1.2 ± 1.0 | 1.9 ± 1.8 |
| HBGI, all | 7.7 ± 4.3 | 0.3 ± 0.5 |
| Male | 7.7 ± 4.5 | 0.4 ± 0.6 |
| Female | 7.6 ± 4.2 | 0.3 ± 0.4 |
| Interday CV (%), all | 37.1 ± 8.6 | 16.6 ± 5.4 |
| Male | 37.3 ± 9.0 | 16.2 ± 5.2 |
| Female | 36.9 ± 8.2 | 17.0 ± 5.6 |
| Intraday CV (%), all | 34.1 ± 7.8 | 16.6 ± 5.4 |
| Male | 34.0 ± 8.3 | 16.2 ± 5.2 |
| Female | 34.1 ± 7.3 | 17.0 ± 5.6 |

**Supplementary Table S3:** Training and model hyperparameters used in GluLLM experiments.

| **Parameter** | **Value** |
| --- | --- |
| **Optimisation** | |
| batch size | 32 |
| learning rate | 1 × 10^−4^ |
| **Adapter configuration** | |
| dropout | 0.1 |
| mlp_hidden_dim | 256 |
| mlp_hidden_layers | 2 |
| mlp_activation | tanh |

# References

[1] S. Del Favero, A. Facchinetti, C. Cobelli, A glucose-specific metric to assess predictors and identify models, *IEEE Transactions on Biomedical Engineering* 59 (5) (2012) 1281–1290.
